# Cancer incidence and mortality for all causes in HIV-infected patients over a quarter century: a multicentre cohort study

**DOI:** 10.1186/s12889-015-1565-0

**Published:** 2015-03-12

**Authors:** Elena Raffetti, Laura Albini, Daria Gotti, Daniela Segala, Franco Maggiolo, Elisa di Filippo, Annalisa Saracino, Nicoletta Ladisa, Giuseppe Lapadula, Chiara Fornabaio, Filippo Castelnuovo, Salvatore Casari, Massimiliano Fabbiani, Piera Pierotti, Francesco Donato, Eugenia Quiros-Roldan, MASTER Cohort

**Affiliations:** Unit of Hygiene, Epidemiology and Public Health, University of Brescia, Brescia, Italy; University Division of Infectious and Tropical Diseases, University of Brescia, Brescia, Italy; Department of Infectious Diseases of Nuovo Polo Ospedaliero S.Anna di Cona, Ferrara, Italy; Department of Infectious Diseases of Papa Giovanni XXIII Hospital, Bergamo, Italy; Department of Infectious Diseases of Polyclinic of Bari, University of Bari, Bari, Italy; Department of Infectious Diseases, San Gerardo de’ Tintori” Hospital, Monza, Italy; Clinic of Infectious Diseases of Istituti Ospitalieri of Cremona, Cremona, Italy; Hospital Division of Infectious and Tropical Diseases, Spedali Civili Hospital, Brescia, Italy; Institute of Clinical Infectious Diseases of Polyclinic A. Gemelli, University of Sacred Heart, Rome, Italy; Department of Infectious Diseases of SM, Annunziata Hospital of Florence, Florence, Italy

**Keywords:** HIV-infection, Cancer, Mortality, Epidemiology

## Abstract

**Background:**

We aimed to assess cancer incidence and mortality for all-causes and factors related to risk of death in an Italian cohort of HIV infected unselected patients as compared to the general population.

**Methods:**

We conducted a retrospective (1986–2012) cohort study on 16 268 HIV infected patients enrolled in the MASTER cohort. The standardized incidence ratios (SIRs) and standardized mortality ratios (SMRs) were computed using cancer incidence rates of Italian Cancer Registries and official national data for overall mortality. The risk factors for death from all causes were assessed using Poisson regression models.

**Results:**

1,195 cancer cases were diagnosed from 1986 to 2012: 700 AIDS-defining-cancers (ADCs) and 495 non-AIDS-defining-cancers (NADCs). ADC incidence was much higher than the Italian population (SIR = 30.8, 95% confidence interval 27.9-34.0) whereas NADC incidence was similar to the general population (SIR = 0.9, 95% CI 0.8-1.1). The SMR for all causes was 11.6 (11.1-12.0) in the period, and it decreased over time, mainly after 1996, up to 3.53 (2.5-4.8) in 2012. Male gender, year of enrolment before 1993, older age at enrolment, intravenous drug use, low CD4 cell count, AIDS event, cancer occurrence and the absence of antiretroviral therapy were all associated independently with risk of death.

**Conclusions:**

In HIV infected patients, ADC but not NADC incidence rates were higher than the general population. Although overall mortality in HIV infected subjects decreased over time, it is about three-fold higher than the general population at present.

## Background

The introduction and widespread use of combination antiretroviral therapy (cART) in the mid-1990s has dramatically improved the health status of HIV-infected individuals, whose life expectancy is now similar to that of the general population [1-7]. The progressive aging of HIV-positive patients due to the decrease of AIDS-related deaths has led to increasing occurrence of other chronic disease, such as cancer [8-11], which is now the leading cause of death among these patients [3-5]. However, despite the improvement in overall survival, total mortality still differs between HIV infected and non-infected subjects [2,12].

Limited data are available on cancer incidence and mortality in Italian HIV-infected subjects and most of them were provided by a record linkage between national AIDS registry and cancer registries performed at the beginning of cART era [13-17].

In recent years, some international pooling-cohort studies have described cancer incidence and mortality after diagnosis of cancer in HIV-infected subjects [4,5,11,18]. However, Italian HIV-infected patients show different characteristics with respect to patients included in European and US cohorts, particularly a higher likelihood of risk-taking behaviors for cancer, such as tobacco smoking, alcohol abuse and hepatitis B (HBV) and C (HCV) co-infections [19]. Indeed, recently, the ART Cohort Collaboration found substantial differences in mortality rates between HIV subjects living in Europe and in North America due to various factors, including behavioral characteristics and co-morbidities such as HCV infection [20].

The aims of this study were: (i) to describe cancer incidence and mortality from all causes in a large unselected Italian cohort of HIV-infected patients from 1986 to 2012, and (ii) to assess the factors associated with risk of death.

## Methods

### MASTER cohort and study population

The Italian MASTER cohort is a hospital-based multicenter, open, dynamic cohort established in the mid-1990s, with retrospective patients’ enrolment from 1986 to 1997 and prospective recruitment subsequently. At present, the cohort includes about 24,500 HIV-infected adults, aged 18 years or older, in care at eight Italian hospitals [19]. For this study, two centers were excluded because did not codify cancer cases. Enrollment in MASTER is independent of the HIV disease stage, degree of immunosuppression or use of antiretroviral therapy. Clinical data are recorded every three/four months in an electronic chart (NetCare™ or Health&Notes™). Data merging and cleaning are performed at a central level every six months.

In this study, we have conducted a retrospective cohort study from January 1986 to September 2012 on HIV-infected patients for which the following data were available: date of first HIV positive test or cohort-entry, date of death for patients died during the study period, date of last visit for patients still alive or lost to follow-up and at least one CD4 cell count. Patients with cancer diagnosis before enrolment in the cohort were excluded. Patients without data recorded for one year and over were considered lost to follow-up. We defined cART regimen the treatment with 3 or more antiretroviral drugs belonging to at least two different drug classes, and “sub-optimal cART” any other regimen.

In this study we retrieved from the MASTER electronic database: gender, age at enrolment, country of origin, HIV exposure group, period of enrolment in the cohort and Hepatitis C or B co-infection. Furthermore, values of HIV-RNA, CD4 cell count, AIDS event and cART at enrolment and every year were also collected.

### Cancer diagnoses and deaths

Cancer diagnoses were collected from medical records and verified through a standardized process, including detailed record abstraction and checking. Only incident cancers occurring during the follow-up were included, whereas cancer recurrences and metastases were excluded. Cancer type and site were coded according to the International Classification of Diseases (ICD), 9th revision, and the International Classification of Diseases, 10th revision [21]. Malignancies were defined as AIDS-defining-cancers (ADCs), including non-Hodgkin’s lymphoma, Kaposi’s sarcoma and invasive cervical carcinoma, and non-AIDS-defining-cancers (NADCs), including all the other types of cancers. Vital status and date of death were ascertained through clinical charts, and through a record-linkage with Local Health Authority mortality registers in about one third of patients.

### Statistical analysis

Patients were classified into 3 groups according to cancer occurrence: i) patients who developed ADC; ii) patients who developed NADC; iii) patients without cancer diagnosis (free-cancer patients). Quantitative variables were compared by the Mann–Whitney test; the χ^2^test was used to assess independence between qualitative variables.

The follow-up was determined from the date of enrollment in the cohort to the end of the observation period. For computing cancer incidence, the end of the period was established as: the date of cancer diagnosis, September 30th, 2012 (December 31th 2009 for SIR analysis), death, or last follow-up visit for patients lost to follow-up, whichever occurred first. For mortality analysis, the observation period finished at the earliest of September 30th, 2012, death or last follow-up visit for patients lost to follow-up. Cancer incidence rates (IRs) were computed for ADC and NADC, separately, whereas mortality rates were calculated for three groups: patients with ADC, NADC, or without cancer. Incidence and mortality rates were computed for two periods according to cohort entry year: before cART-era (before 1998, i.e. up to 1997) and during the cART-era (in and after 1998). All the rates were calculated dividing observed cases by the corresponding person-years at risk, standardized for gender and age using the direct method with the European population as the standard and truncated at 65 years-old. The rates were expressed per 1,000 person-years (1000 PYs). Cancer incidence in this cohort was compared with that observed in the Italian general population computing the standardized incidence ratios (SIRs) and their corresponding 95% confidence intervals (95% CIs), with the Byar’s approximation of Poisson model, using the Italian general population gender- and age-specific rates provided by Italian Cancer Registries. Since cancer incidence rates vary substantially from one region to another in Italy and the composition of HIV cohort is highly unbalanced (15.6% from South Italy and 84.4% from North Italy), 5 population cancer registries (Torino, Varese, Ferrara, Latina, Ragusa) with data collected in 1986–2009 period were selected and weighted according to area of residence of HIV subjects for a proper comparison. SIRs were calculated until 2009, since data from cancer registries were updated to that year.

Similarly, the standardized mortality ratios (SMRs) for all causes were computed as the ratio of observed to the expected numbers of deaths, and their corresponding 95% CIs were estimated using the Byar’s approximation of Poisson model. For this purpose, we used the sex- and age-standardized mortality rates of the Italian general population in 1986–2003 and 2006–2011 provided by the Istituto Superiore di Sanità. For convenience, we extended the mortality rates in 2003 and 2006 to 2004 and 2005, respectively, and the mortality rate in 2011 to 2012. Three analyses of the SMRs were performed: i) the temporal trend in 1986–2012; ii) the global SMRs, calculated for the overall cohort and iii) SMRs stratified for gender and age. To evaluate time trend of SMR, we fitted a regression model with fractional polynomial terms for year as independent variable, using the Akaike’s information criterion to choose the number of degrees of fractional polynomial terms.

The association of each investigated variable with all-cause mortality was tested by univariate and multivariate analysis using time dependent Poisson regression models with study period divided into intervals of 1-year duration, which provided estimates of relative risks (RRs), their 95% CIs and p-values. Gender, age at enrolment, intravenous drug use as risk factors, hepatitis C or B virus co-infection and year of enrolment were included in the model as fixed covariates. Cancer occurrence, AIDS events (ADC was not considered as an AIDS event), CD4 cell count and antiretroviral therapy were included as time-dependent covariates.

Finally, to evaluate the causal effect of cART on risk of death, we used a marginal structural statistical model [22], with cancer occurrence, AIDS events, and CD4 cell count as time-dependent covariates. Inverse-probability-of-treatment weights were determined on the basis of the propensity score estimated by multinomial logistic regression with antiretroviral therapy as the dependent variable, categorized as i) cART: (≥3 antiretroviral drugs from at least 2 different classes), ii) sub-optimal-cART (any other regimen) ,and iii) no therapy.

The selection of variables for the most parsimonious models was performed using a backward stepwise procedure, with p = 0.20 for retaining each variable in the models. However, age and gender were also retained in all the models as possible confounders.

All statistical tests were two-sided, assumed a level of significance of 0.05 and were performed using Stata 12 software (Stata Statistics/Data Analysis 12.0 - Stata Corporation, College Station, TX, USA).

### Ethics procedures

At first visit, patients provide written informed consent for including their clinical and biological data in the MASTER database for scientific purpose. Data submitted by the participating clinics to the data center are anonymized. The study was approved by the Ethical Committees of the Hospital Spedali Civili of Brescia (Coordinating Centre) and of the following Institutions: University Hospital of Ferrara, Ferrara; AO Papa Giovanni XXIII, Bergamo; University of Bari, Bari; San Gerardo de’ Tintori” Hospital, Monza; Hospital of Cremona, Cremona; “Santa Maria Annunziata” Hospital, Firenze; University of Sacred Heart, Roma.

## Results

### Cancer incidence

A total of 16,268 HIV infected patients were enrolled, contributing 134,291 person-years (median 6.2 years of follow-up). The cumulative probability of loss to follow-up at 3 years after enrolment was 22.7% (95% CI 22.0%-23.3%). A total of 1,195 cancer cases were diagnosed from 1986 to 2012 in 1,159 patients: 700 (60%) ADCs and 495 (40%) NADCs, including 36 individuals with both ADC and NADC. The patients’ characteristics at enrolment are shown in Table 1. At baseline, patients with NADC, respect to those with ADC and those without cancer, were older, had a higher proportion of intravenous drugs users (IDUs), HBV or HCV co-infection, and an intermediate CD4 cell count mean.

**Table 1:** **Patients’ characteristics at enrolment according to cancer-categories**

**Variables**	**Categories**	**Total**	**ADC**	**NADC**	**Without cancer**	**P value**
**Total**		16268	688	471	15109	
**Gender**	**Male**	12116 (74.5)	549 (79.8)	360 (76.4)	11207 (74.2)	**0.003**
	**Female**	4152 (25.5)	139 (20.2)	111 (23.6)	3902 (25.8)	
**Age at enrolment (years)**	**18-34**	9797 (60.2)	369 (53.6)	228 (48.4)	9200 (20.9)	**<0.001**
	**35-44**	4278 (26.3)	189 (27.5)	121 (25.7)	3968 (26.3)	
	**45-54**	1538 (9.5)	91 (13.2)	71 (15.1)	1376 (9.1)	
	**55-64**	513 (3.1)	36 (5.2)	41 (8.7)	436 (2.9)	
	**>65**	142 (0.9)	3 (0.4)	10 (2.1)	129 (1.0)	
	**Mean (SD)**	34.2 (9.8)	35.6 (10.3)	37.8 (11.9)	34.1 (9.7)	**<0.001**
**Year of enrolment**	**1986-1989**	2275 (14.0)	79 (16.8)	135 (19.6)	2061 (13.6)	**<0.001**
	**1990-1993**	2835 (17.4)	99 (21.0)	121 (17.6)	2615 (17.3)	
	**1994-1997**	2465 (15.1)	98 (20.8)	137 (19.9)	2230 (14.8)	
	**1998-2001**	2522 (15.5)	73 (15.5)	101 (14.7)	2348 (15.5)	
	**2002-2005**	2539 (15.6)	70 (14.9)	108 (15.7)	2361 (15.6)	
	**2006-2012**	3632 (22.3)	52 (11.0)	86 (12.5)	3494 (23.1)	
**IDUs**	**No**	8374 (51.5)	363 (52.8)	200 (42.5)	7811 (51.7)	**<0.001**
	**Yes**	7894 (48.5)	325 (47.2)	271 (57.5)	7298 (48.3)	
**HBV/HCV co-infection**	**No**	8374 (51.5)	363 (52.8)	200 (42.5)	7811 (51.7)	**<0.001**
	**Yes**	7894 (48.5)	325 (47.2)	271 (57.5)	7298 (48.3)	
**CD4 cell count , cell/mm3**	**0-49**	1151 (7.1)	95 (13.8)	28 (5.9)	1028 (6.8)	**<0.001**
	**50-99**	940 (5.8)	59 (8.6)	31 (6.6)	850 (5.6)	
	**100-199**	2038 (12.5)	136 (19.8)	70 (14.9)	1832 (12.1)	
	**200-349**	3464 (21.3)	144 (20.9)	100 (21.2)	3220 (21.3)	
	**350-499**	3346 (20.6)	110 (16.0)	107 (22.7)	3129 (20.7)	
	**≥500**	5329 (32.8)	144 (20.9)	135 (28.7)	5050 (33.4)	
	**Mean (SD)**	416.8 (298.6)	316.7 (272.6)	401.8 (300.8)	421.8 (298.9)	**<0.001**
**Log10 HIVRNA, copies/mL**	**Mean (SD)**	3.1 (1.4)	3.3 (1.5)	3.0 (1.4)	3.1 (1.4)	NS
**Treatment-naïve**	**No**	14179 (87.2)	610 (88.7)	412 (87.5)	13157 (87.1)	NS
	**Yes**	2089 (12.8)	78 (11.3)	59 (12.5)	1952 (12.9)	

Patients with both ADC and NADC diagnosis (n = 36) were considered as ADC or NADC on the basis of which first occurred cancer.

*Abbreviations*: *ADC* AIDS-defining-cancer, *NADC* non-AIDS-defining-cancer, *SD* standard deviation, *NS* not statistically significant (p > 0.05), *IDUs* intravenous drug users, *HBV/HCV co-infection* hepatitis B virus/hepatitis C virus positivity.

At cancer diagnosis, patients with NADC were older than those with ADC (median age of 45 [IQR 39–53] vs. 39 [IQR 33–46] years, p <0.001) and showed a higher CD4 cell count (median of 334 [IQR 180–482] vs. 145 [IQR 46–329] cell/mm^3^, p <0.001) (data not shown in Table).

The comparison of sex- and age-standardized incidence rates (IRs) for all ADCs and for all NADCs from before to after 1998 showed a significant decrease of the IRs for ADC, from 14.7 (95% CI 9.8-19.5) to 4.2 (3.7-4.8) per 1000 PYs (p = 0.002), but an increase of the IRs for NADC from 3.4 (0.5-6.3) to 4.6 (3.9-5.3) per 1000 PYs (p = 0.01). Overall incidence of ADCs was higher than the Italian general population with SIR = 30.8 (27.9-34.0), with a significant decrease from 67.7 (59.3-77.1) to 30.8 (27.9-34.0), before and after 1998, respectively. The global incidence of NADCs was similar to that detected in the Italian general population with SIR = 0.9 (0.8-1.1), both before and after 1998, with SIR = 0.9 (0.6-1.2) and SIR = 0.9 (0.8-1.1), respectively (data not shown in Table).

The SIRs of specific cancers in the whole period are shown in Table 2. Very high SIRs were found, in decreasing order, for Kaposi’s sarcoma (SIR = 168.5), penis (SIR = 28.0), non-Hodgkin lymphoma (SIR = 24.5), tongue and lingual tonsil (SIR = 22.1), vagina and vulva (SIR = 22.1), cervix (SIR = 17.9), liver (SIR = 10.6) and Hodgkin lymphoma (SIR = 11.8).

**Table 2:** **Observed and expected cancers among the HIV-infected patients as compared to the general population**

**Observational-time**	**1986-2009**		
**Cancer type or site (ICD-10)**	**Observed (n)**	**Expected (n)**	**SIR (95% CI)**
**AIDS-defining-cancer**			
**Kaposi’s sarcoma** (C46)	**287**	**1.7**	**168.5 (149.6-189.2)**
**Cervix Uteri** (C53)^	**41**	**2.3**	**17.9 (12.8-24.2)**
**Non-Hodgkin lymphoma** (C82-5, C96)	**313**	**12.8**	**24.5 (21.8-27.3)**
**Non-AIDS-defining-cancer**			
**Tongue and lingual tonsil** (C01-C02)	**5**	**0.2**	**22.1 (7.1-51.5)**
Stomach (C16)	9	6.5	1.4 (0.6-2.6)
**Colon** (C18)	**32**	**14.2**	**2.2 (1.5-3.2)**
**Rectum-Anus (C19-21)**	**24**	**7.7**	**3.1 (2.0-4.7)**
**Liver, primary** (C22)	**61**	**5.8**	**10.6 (8.1-13.6)**
**Larynx** (C32)	**9**	**3.3**	**2.8 (1.3-5.3)**
**Lung, bronchus and trachea** (C33-34)	**29**	**19.5**	**1.5 (1.0-2.1)**
Melanoma (C43)	14	12.9	1.1 (0.6-1.8)
Breast (C50)	32	24.7	1.3 (0.9-1.8)
**Vagina and vulva** (C51-52, 57)	**5**	**0.2**	**22.1 (7.1-51.5)**
**Penis** (C60)	**8**	**0.3**	**28.0 (12.1-55.2)**
Prostate (C61)	8	16.2	0.5 (0.3-1.0)
**Testis** (C62)	**19**	**7.9**	**2.4 (1.4-3.7)**
Kidney (C64-66, C68)	9	8.9	1.0 (0.5-1.9)
Bladder (C67)	6	13.4	0.4 (0.2-1.0)
Brain and CNS (C70-72)	4	6	0.7 (0.2-1.7)
Thyroid (C73)	7	11.3	0.6 (0.2-1.3)
**Hodgkin lymphoma** (C81)	**59**	**5**	**11.8 (9.0-15.2)**
Myeloid leukaemia (C92)	4	2.7	1.5 (0.4-3.7)

**^**Cervix uteri was included in AIDS-defining-cancer after 1993. In bold are reported cancers with statistical significant SIRs (p < 0.05). We did not performed SIR for cancers with less than 4 cases.

*Abbreviations*: *ICD* international classification of diseases, *SIR* standardized incidence ratio, *95% CI* 95% confidence interval.

### Mortality

A total of 2,847 (17%) patients died during follow-up (2,191 patients before and 656 after 1998), with a mortality rate of 19.3 per 1,000 PYs, from 57.3 to 13.3 per 1,000 PYs, before and after 1998, respectively. The sex- and age-standardized mortality rates showed a 2–3 fold decline in both patients without cancer and those with ADC, but a 2-fold increase among individuals with NADC in the period after, with respect to before 1998 (Table 3).

**Table 3:** **Age-standardized mortality rate according to cancer-categories in period 1986–2012**

	**Age-standardized mortality rate (95% CI)**	
	**Before 1998**	**After 1998**
**ADC**	96.1 (57.5-113.5)	29.1 (23.0-35.3)
**NADC**	14.3 (0.0-35.3)	27.5 (22.5-32.6)
**Without cancer**	56.7 (45.4-68.0)	11.5 (10.5-12.6)

Patients with both ADC and NADC diagnosis (n = 36) were considered as ADC or NADC on the basis of which cancer group first occurred.

*Abbreviations*: *ADC* AIDS-defining-cancer, *NADC* non-AIDS-defining-cancer, *95% CI* 95% confidence interval.

**Figure 1:**
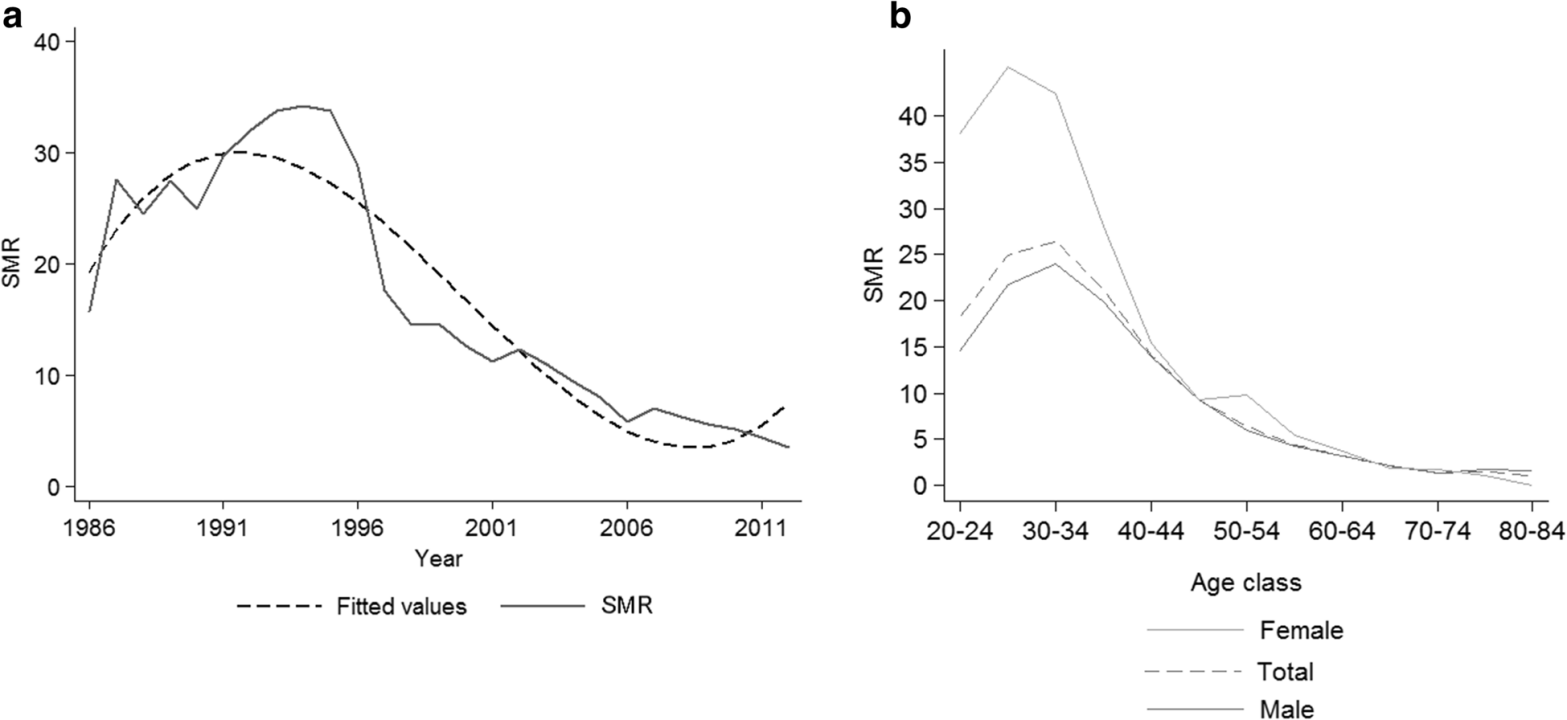
**a) Standardized mortality ratio (SMR) from 1986 to 2012); b) SMR stratified by gender-and age-class.**

The HIV-infected patients had a higher mortality than the Italian general population, showing a global SMR of 11.6 (11.1-12.0), higher in females than in males (16.0 [14.7-17.4] *vs*. 10.8 [10.4-11.3]) (data not shown in Table). The SMR for all causes showed a steady decrease after 1996, up to 2006, and it was stable afterwards, though mortality was still higher than the general population in 2012, with SMR = 3.53 (2.5-4.8) (Figure 1a). The gender-and age-stratified SMRs are shown in Figure 1b. The SMRs decreased with increasing age, from 18.3 (14.3-23.2) in those aged 20–24 years to 2.1 (1.5-2.9) in those aged 65–69 years, and 1.3 (0.8-1.2) in patients aged 70 years or older (Figure 1b). Among patients of 20–39 years of age, women showed higher SMRs than men, from 38.2 (24.2-57.3) and 14.6 (10.7-19.4) in subjects aged 20–24 to 28.1 (23.2-33.6) and 19.9 (18.2-21.8) in subjects aged 35–39, in females and males, respectively.

### Predictors of mortality for all causes

We evaluated the factors associated with death from all causes by univariate and multivariate analysis, including also cancer occurrence as independent variable. Both analyses showed a higher risk of death for male gender, year of enrolment before 1993, older age at enrolment, intravenous drug use and the following variables included as time-dependent covariates: low CD4 cell count, AIDS event, cancer occurrence and the absence of antiretroviral therapy (Table 4). However this model was not proper to evaluate the cART effect, because of non-independence of cART, CD4 cell count and AIDS event. Therefore, we estimated the causal effect of cART using marginal structural models: cART was strongly protective against mortality with HR = 1.79 (1.49-2.16, p < 0.001) for sub-optimal-cART (<3 drugs for at least 2 classes) and HR = 2.09 (1.83-2.39, p < 0.001) for no therapy as respect to having received optimal cART (data not shown in Table).

**Table 4:** **Univariate and multivariate Poisson regression with death for all-causes as the outcome**

		**Univariate model**		**Multivariate model**	
**Variables**	**Categories**	**RR**	**95% CI**	RR*	**95% CI**
**Gender**	**Male vs Female**	1.55	(1.42-1.70)	1.24	(1.12-1.36)
**Age at enrolment (years)**	**18-34**	Ref.		Ref.	
	**35-44**	0.96	(0.88-1.06)	1.53	(1.36-1.71)
	**45-54**	1.28	(1.12-1.47)	2.41	(2.05-2.83)
	**55-64**	1.56	(1.27-1.92)	2.66	(2.13-3.33)
	**≥65**	3.06	(2.24-4.19)	5.41	(3.89-7.52)
**Year of enrolment**	**1986-1989**	2.41	(1.99-2.91)	1.55	(1.25-1.94)
	**1990-1993**	2.23	(1.84-2.70)	1.43	(1.15-1.78)
	**1994-1997**	1.63	(1.34-1.98)	0.89	(0.71-1.10)
	**1998-2001**	1.23	(1.00-1.51)	1.11	(0.90-1.36)
	**2002-2005**	1.01	(0.81-1.27)	1.00	(0.80-1.26)
	**2006-2012**	Ref.		Ref.	
**IDUs**	**Yes vs No**	1.76	(1.62-1.90)	1.54	(1.40-1.70)
**AIDS-event**	**Yes vs No**	17.55	(16.22-18.99)	3.75	(3.43-4.11)
**HBV/HCV co-infection**	**Yes vs No**	0.95	(0.89-1.03)		
**CD4 cell count, cell/mm3**	**0-49**	44.92	(37.90-53.25)	4.10	(3.62-4.65)
	**50-99**	17.58	(14.48-21.33)	2.73	(2.36-3.16)
	**100-199**	7.78	(6.45-9.39)	1.83	(1.62-2.08)
	**200-349**	3.02	(2.45-3.67)	1.47	(1.31-1.65)
	**350-499**	1.64	(1.32-2.04)	1.15	(1.01-1.30)
	**≥500**	Ref.		Ref.	
**Antiretroviral therapy**	**cART**	Ref.		Ref.	
	**Subobtimal cART**	3.26	(2.91-3.65)	2.18	(1.95-2.44)
	**No therapy**	2.14	(1.96-2.33)	8.19	(7.23-9.27)
**Cancer occurrence**	**Without cancer**	Ref.		Ref.	
	**ADC**	14.60	(12.51-17.03)	1.22	(1.08-1.38)
	**NADC**	10.48	(8.49-12.93)	1.29	(1.12-1.50)

Time-dependent Poisson regression models, with observational period divided into intervals of 1-year duration. Gender, age at enrolment, year of enrolment, intravenous drug use as risk factors and hepatitis C or B virus co-infection were included in the model as fixed covariates. CD4 cell count, AIDS events (ADC was not considered as an AIDS event), antiretroviral therapy and cancer occurrence were included as time-dependent covariates, considering at enrolment and every year.

*Adjusted for all the variables in the model.

*Abbreviations*: *RR* relative risk, *95% CI* 95% confidence interval, *IDUs* intravenous drug users, *HBV/HCV co-infection* hepatitis B virus/hepatitis C virus positivity, *cART* combined antiretroviral therapy.

## Discussion

This study describes AIDS-defining and non-AIDS-defining cancer incidence and mortality for all causes in a large multicenter Italian cohort of HIV/AIDS patients, over a quarter century, up to 2012. As regards ADC, we found that both incidence rates (IRs) and observed/expected incidence ratios (SIRs) decreased of about 2–3 fold, from before to after 1998. In contrast, for NADC we observed a significant increase of the IRs but no variation of SIRs from before to after 1998. As regards mortality, the overall death rates decreased of 3–4 fold from before to after 1998. The observed/ expected ratio of death for all causes (SMR) declined after 1996 up to 2012, though it was still higher than that observed in the general population, more in females than males, in 2012.

Inconsistent estimates of cancer incidence in HIV subjects were reported in various studies in last decades. These discrepancies can be attributed to the different populations or subgroups investigated, such as HIV-infected [2,4,5,8] or AIDS [10,13-16] patients or subjects at high risk of exposure to oncogenic viruses (HCV/HBV co-infected for liver cancer [9] or homosexual men for HPV-related cancer [23]. However, most of these findings were in agreement with ours, showing a decrease in the incidence of ADCs and an increase of NADCs after cART introduction [24,25]. Indeed cART determines immune reconstitution and increases HIV-infected patients life-expectancy [1], so as they are at risk of developing age-related cancers as much as the general population. However, the risk of developing some NADCs still remains greater than in non-HIV-infected subjects especially virus-related cancer such as tongue and lingual tonsil, rectum-Anus, liver, larynx, vagina and vulva, penis cancer and Hodgkin lymphoma.

As regards overall mortality of the cohort, our findings are in agreement with previous studies that reported a substantial reduction in mortality rates among HIV-infected subjects after 1998, mainly attributed to the decline of deaths for AIDS-related conditions, thanks to cART [1,3,18]. The global SMR in our study is slightly higher than the values found in other HIV seropositive cohorts in Italy and other countries [2,26-32], probably because of the higher prevalence of IDUs in our cohort. In fact, overall mortality among IDUs is about 10 times higher than the general population [33] and several HIV seropositive IDUs still have a relatively poor prognosis, due to drug use by itself, mental health illness, HCV/HBV co-infection or poor adherence to cART [26,27,33-36].

The SMR decreased with age, from 20–24 to 50–54 years, with mortality rates close to the general population’s ones in HIV-infected individuals of 70 years and older, as previously described [2,7,12,18,29]. In fact, young HIV-infected individuals have a higher risk of death than the general population due to the presence of HIV-infection itself, higher prevalence of intravenous drug use, tobacco smoking or heavy alcohol consumption, HCV/HBV co-infection or low compliance to therapy [33-36]. On the other hand, older HIV-positive individuals are more strictly monitored than individuals of the same age in the general population, getting earlier diagnosis and treatment for possible co-morbidities [2]. We found that women younger than 40 years had higher SMRs than men, similarly to that reported in other cohorts [2,7,12,35], maybe because they had less favorable socio-economic or life-style factors or a delayed first HIV test [2,18]. Further studies are necessary to find out the causes of this gender difference.

In the multivariate analysis, we found that CD4 cell count, AIDS event, cancer diagnosis occurrence, and the underuse or the absence of cART were associated with overall mortality. The protective effect of cART on risk of death was also confirmed by the analysis with a marginal structural model. These findings support the widespread view that strict cART adherence and early diagnosis of HIV infection are important for reducing the risk of death among HIV-infected individuals [37].

This study has various points of strength. First, the MASTER cohort provides an unbiased “real world” scenario of the clinical practice and epidemiological pattern of HIV infection and AIDS disease throughout Italy, from the beginning of HIV epidemic (1986) to today. Second, this study has a long-term follow-up, longer than most HIV cohort studies published so far, which analyzed mortality only after 1998 [3-7,18].

This study has some limitations, too. First, as regards cancer incidence, we did not perform a record-linkage with Italian Cancer Registries, so some cancer cases may have not been captured in this analysis, especially NADCs. Therefore, the NADC incidence rates found in our study may have underestimated the true values. However, we are confident that cancer records collected by the HIV clinical personal were sufficiently accurate to minimize possible loss of cases. Indeed, in a previous study that we performed in Brescia using a record linkage with a population base cancer registry, we found similar results showing a moderately higher incidence of overall NADCs with SIR = 1.6 (1.4–1.9) [8].

As regards the vital status of subjects during and at the end of the follow-up, we performed a record-linkage of our data with those from mortality registries of Local Health Authorities in some centers for about 30% of the subjects and we found that the HIV original database included 793 and the record linkage with Local Health Authorities allow us to add 23, corresponding to 3% of the total deaths, thus producing a possible modest underestimate of all-cause mortality. Therefore, we are confident that the deaths recorded were also sufficiently complete in the others centers.

Second, we used the cART data concerning drugs prescriptions but did not consider patients’ adherence to therapy. As a consequence, the positive prognostic value of cART may be also higher than that we found in our study.

In conclusion, we found a dramatic reduction of incidence rates of ADCs, which provides support for the hypothesis of positive clinical effect of cART on the long term, and no variation of NADC incidence with respect to the general population over time. Overall mortality rates decreased over time, though they are still higher than those observed in the general population at present.

## Abbreviations

ADC: AIDS-defining-cancer
cART: Combination antiretroviral therapy
HBV: Hepatitis B virus infection
HCV: Hepatitis C virus infection
ICD: International Classification of Diseases
IDU: Intravenous drugs user
IR: Incidence rates
NADC: Non-AIDS-defining-cancer
RR: Relative risk
SIR: Standardized incidence ratio
SMR: Standardized mortality ratio
95% CI: 95% confidence interval

## References

[1] Guaraldi G, Cossarizza A, Franceschi C, Roverato A, Vaccher E, Tambussi G (2014). Life expectancy in the immune recovery era: the evolving scenario of the HIV epidemic in northern Italy. JAIDS.

[2] Galli L, Spagnuolo V, Salpietro S, Gianotti N, Cossarini F, Lazzarin A (2012). Mortality of HIV-infected patients with or without cancer: comparison with the general population in Italy. Antivir Ther.

[3] Weber R, Ruppik M, Rickenbach M, Spoerri A, Furrer H, Battegay M (2013). Decreasing mortality and changing patterns of causes of death in the Swiss HIV Cohort Study. HIV Med.

[4] Smith CJ, Ryom L, Weber R, Morlat P, Pradier C, Reiss P (2014). Trends in underlying causes of death in people with HIV from 1999 to 2011 (D:A:D): a multicohort collaboration. Lancet.

[5] Antiretroviral Therapy Cohort Collaboration (2010). Causes of death in HIV-1-infected patients treated with antiretroviral therapy,1996-2006: collaborative analysis of 13 HIV cohort studies. Clin Infect Dis.

[6] McManus H, O'Connor CC, Boyd M, Broom J, Russell D, Watson K (2012). Long-term survival in HIV positive patients with up to 15 Years of antiretroviral therapy. PLoS One.

[7] Simmons R, Ciancio B, Rice BD KM, Delpech VC (2013). Ten-year mortality trends among persons diagnosed with HIV infection in England and Wales in the era of antiretroviral therapy: AIDS remains a silent killer. HIV Med.

[8] Calabresi A, Ferraresi A, Festa A, Scarcella C, Donato F, Vassallo F (2013). Incidence of AIDS-defining cancers and virus-related and non-virus-related non-AIDS-defining cancers among HIV-infected patients compared with the general population in a large health district of northern Italy, 1999–2009. HIV Med.

[9] Albini L, Calabresi A, Gotti D, Ferraresi A, Festa A, Donato F (2013). Burden of Non-AIDS-defining and Non-virus-related cancers among HIV-infected patients in the combined antiretroviral therapy Era. AIDS Res Hum Retroviruses.

[10] Simard EP, Pfeiffer RM, Engels EA (2012). Mortality due to cancer among people with AIDS: a novel approach using registry-linkage data and population attributable risk methods. AIDS.

[11] Smith C, Sabin CA, Lundgren JD, Thiebaut R, Weber R, Data Collection on Adverse Events of Anti-HIV drugs (D:A:D) Study Group (2010). Factors associated with specific causes of death amongst HIV-positive individuals in the D:A:D Study. AIDS.

[12] Van Sighem A, Danner S, Ghani AC, Gras L, Anderson RM, de Wolf F (2005). Mortality in patients with successful initial response to highly active antiretroviral therapy is still higher than in non-HIV-infected individuals. J Acquir Immune Defic Syndr.

[13] Dal Maso L, Suligoi B, Franceschi S, Braga C, Buzzoni C, Polesel J (2014). Survival after cancer in Italian persons with AIDS, 1986–2005: a population-based estimation. J Acquir Immune Defic Syndr.

[14] Zucchetto A, Suligoi B, De Paoli A, Pennazza S, Polesel J, Bruzzone S (2010). Excess mortality for non–AIDS-defining cancers among people with AIDS. Clin Infect Dis.

[15] Polesel J, Franceschi S, Suligoi B, Crocetti E, Falcini F, Guzzinati S (2010). Cancer incidence in people with AIDS in Italy. Int J Cancer.

[16] Dal Maso L, Polesel J, Serraino D, Lise M, Piselli P, Falcini F (2009). Pattern of cancer risk in persons with AIDS in Italy in the HAART era. Br J Cancer.

[17] Dorrucci M, Balducci M, Pezzotti P, Sinicco A, Alberici F, Rezza G (1999). Temporal changes in the rate of progression to death among Italians with known date of HIV seroconversion: estimates of the population effect of treatment. Italian HIV Seroconversion Study (ISS). J Acquir Immune Defic Syndr.

[18] Lewden C, Bouteloup V, De Wit S, Sabin C, Mocroft A, Collaboration of Observational HIV Epidemiological Research Europe (COHERE) in EuroCoord (2012). All-cause mortality in treated HIV-infected adults with CD4≥500/mm3 compared with the general population: evidence from a large European observational cohort collaboration. Int J Epidemiol.

[19] MASTER Cohort. website http://www.mastercohort.it. Last access 23th October 2014.

[20] May MT, Hogg RS, Justice AC, Shepherd BE, Costagliola D, Ledergerber B (2012). Heterogeneity in outcomes of treated HIV-positive patients in Europe and North America: relation with patient and cohort characteristics. Int J Epidemiol.

[21] World Health Organization. International Classification of Disease (ICD)-10 http://www.who.int/classifications/icd/en/. Last access 23th October 2014.

[22] Robins JM, Hernán MA, Brumback B (2000). Marginal structural models and causal inference in epidemiology. Epidemiology.

[23] Ramqvist T, Dalianis T (2010). Oropharyngeal cancer epidemic and humanpapillomavirus. Emerg Infect Dis.

[24] Cobucci RN, Lima PH, de Souza PC, Costa VV, Cornetta MD, Fernandes JV (2015). Assessing the impact of HAART on the incidence of defining and non-defining AIDS cancers among patients with HIV/AIDS: A systematic review. J Infect Public Health.

[25] Vaccher E, Serraino D, Carbone A, De Paoli P (2014). The evolving scenario of non AIDS-defining cancers: challenges and opportunities of care. Oncologist.

[26] Bhaskaran K, Hamouda O, Sannes M, Boufassa F, Johnson AM, Lambert PC (2008). Changes in the risk of death after HIV seroconversion compared with mortality in the general population. JAMA.

[27] Lewden C, Chene G, Morlat P, Raffi F, Dupon M, Dellamonica P (2007). HIV-infected adults with a CD4 cell count greater than 500 cells/mm3on long term combination antiretroviral therapy reach same mortality rates as the general population. JAIDS.

[28] Zwahlen M, Harris R, May M, Hogg R, Costagliola D, The Antiretroviral Therapy Cohort Collaboration (2009). Mortality of HIV-infected patients starting potent anti-retroviral therapy: comparison with the general population in nine industrialized countries. Int J Epidemiol.

[29] Lewden C, Raffi F, Chene G, Sobel A, Leport C, TheAPROCO Study Group (2001). Mortality in a cohort of HIV-infected adults started on a protease inhibitor-containing therapy - standardization to the general population. JAIDS.

[30] Jaggy C, von Overbeck J, Ledergerber B, Schwarz C, Egger M, Rickenbach M (2003). Mortality in the Swiss HIV Cohort Study (SHCS) and the Swiss general population. Lancet.

[31] Keiser O, Taffe P, Zwahlen M, Battegay M, Bernasconi E, Weber R (2004). All-cause mortality in the Swiss HIV cohort study from 1990 to 2001 in comparison with the Swiss population. AIDS.

[32] Martinez E, Milinkovic A, Buira E, de Lazzari E, León A, Larrousse M (2007). Incidence and causes of death in HIV-infected persons receiving highly active antiretroviral therapy compared with estimates for the general population of similar age and from the same geographical area. HIV Med.

[33] Evans JL, Tsui JI, Hahn JA, Davidson PJ, Lum PJ, Page K (2012). Mortality among young injection drug users in San Francisco: a 10-year follow-up of the UFO study. Am J Epidemiol.

[34] Hernando V, Perez-Cachafeiro S, Lewden C, Gonzalez J, Segura F, Oteo JA (2012). All-cause and liver-related mortality in HIV positive subjects compared to the general population: differences by HCV co-infection. J Hepatol.

[35] Klein MB, Rollet-Kurhajec KC, Moodie EE, Yaphe S, Tyndall M, Walmsley S (2014). Mortality in HIV-hepatitis C co-infected patients in Canada compared to the general Canadian population (2003–2013). AIDS.

[36] van Santen DK, van der Helm JJ, Grady BP, de Vos AS, Kretzschmar ME, Stolte IG (2014). Temporal trends in mortality among people who use drugs compared with the general Dutch population differ by hepatitis C virus and HIV infection status. AIDS.

[37] Denis B, Guiguet M, de Castro N, Mechaï F, Revest M, Mahamat A (2014). Critical importance of long-term adherence to care in HIV infected patients in the cART era: new insights from Pneumocystis jirovecii pneumonia cases over 2004–2011 in the FHDH-ANRS CO4 cohort. PLoS One.

